# Novel Nd-N/TiO_2_ Nanoparticles for Photocatalytic and Antioxidant Applications Using Hydrothermal Approach

**DOI:** 10.3390/ma15196658

**Published:** 2022-09-26

**Authors:** Mir Waqas Alam, N. R. Khalid, Sumaira Naeem, N. A. Niaz, Tanveer Ahmad Mir, Insha Nahvi, Basma Souayeh, Noushi Zaidi

**Affiliations:** 1 Department of Physics, College of Science, King Faisal University, Al Ahsa 31982, Saudi Arabia; 2 Department of Physics, Institute of Physics and Material Science, University of Okara, Okara 56300, Pakistan; 3 Department of Chemistry, University of Gujrat, H. H. Campus, Gujrat 50700, Pakistan; 4 Institute of Physics, Bahauddin Zakariya University, Multan 60800, Pakistan; 5 Laboratory of Tissue/Organ Bioengineering and BioMEMS, Organ Transplant Centre of Excellence, Transplantation Research & Innovation (Dpt)-R, King Faisal Specialist Hospital and Research Centre, Riyadh 11211, Saudi Arabia; 6 Department of Basic Sciences, Preparatory Year Deanship, King Faisal University, Al Ahsa 31982, Saudi Arabia

**Keywords:** Nd-N/TiO_2_, nanoparticles, AB-29 degradation, antioxidant, visible light

## Abstract

In this study, photocatalysis was employed to degrade a wastewater pollutant (AB-29 dye) under visible light irradiation. For this purpose, nitrogen (N)- and neodymium (Nd)-doped TiO_2_ nanoparticles were prepared using the simple hydrothermal method. X-ray diffraction (XRD) revealed an anatase phase structure of the Nd-N/TiO_2_ photocatalyst, whereas properties including the surface morphology, chemical states/electronics structure and optical structure were determined using transmission electron microscopy (TEM), X-ray photoelectron spectroscopy (XPS) and UV–visible (UV–vis.) and photoluminescence (PL) spectroscopies. Photocatalytic testing of the prepared nanomaterials was performed to remove acid blue-29 (AB-29) dye under visible-light exposure. The prepared Nd-N/TiO_2_ nanoparticles demonstrated a superior photocatalytic activity and the decolorization efficiency was about 92% after visible-light illumination for 1 h and 20 min, while N/TiO_2_, Nd/TiO_2_ and TiO_2_ only showed a 67%, 43% and 31% decolorization efficiency, respectively. The enhanced photocatalytic activity of the Nd-N/TiO_2_ photocatalyst was due to a decrease in the electron/hole’s recombination and the increased absorption of TiO_2_ in the visible range. The reusability results showed that the average photocatalytic activity decrease for all the samples was only about 16% after five consecutive cycles, indicating a good stability of the prepared nanomaterials. Moreover, the radical scavenging activity of the prepared nanomaterials was evaluated using the DPPH method. The novel Nd-N/TiO_2_ exhibited a higher antioxidant activity compared to all the other samples.

## 1. Introduction

High rates of industrialization, urbanization and overpopulation result in the production of wastewater on a large scale. Wastewater contains various toxic and harmful substances which are clear threats to human life and the environment [1]. Specially, wastewater contaminants including organic dyes, drugs, pigments, hydrocarbons, etc., are creating permanent damage to ecosystems and the environment [2]. Therefore, wastewater recycling for various objectives requires viable, clean and sustainable technologies for the degradation of toxic water contaminants [3]. For this purpose, the most effective approach is to design an adoptable photocatalyst to decompose the pre-existing environmental pollutants. Heterogeneous photocatalysts such as TiO_2_, ZnO, Fe_2_O_3_, CdS, SnO_2_, V_2_O_5_, g-c_3_N_4_, etc., have been found to effectively photodecompose many harmful and toxic organic pollutants of wastewater [4,5,6,7]. Titanium dioxide has been commonly used in heterogeneous photocatalysis owing to its extraordinary characteristics, namely its low cost, strong oxidizing power and stability in aqueous environments [8]. However, intrinsic TiO_2_ suffers from the lower absorption of visible light, attributable to its broader band gap (~3 eV) and limited photocatalysis activity due to the prompt reunion of charges (electrons and holes) [9,10]. A wide variety of doping techniques have been employed to solve these problems, including doping with transition and noble metals, non-metallic ions and rare earth metals [11,12,13,14,15,16,17]. In particular, the doping of non-metallic ions into TiO_2_ is very easy and is the most active method to achieve solar light absorption in the visible range and higher photocatalysis. It was previously observed that N doping into TiO_2_ systems develops N 2p energy states above the valence band edge of TiO_2_, thus decreasing its band gap and enhancing light utilization in the visible range [18]. Additionally, N doping also reduces the reunion of charges in the N-TiO_2_ system for the better performance of TiO_2_ as a photocatalyst. However, N-TiO_2_ photocatalyst efficiency is still limited because of the low lifetime of the photogenerated charges during photocatalysis. Therefore, doping rare earth metals into TiO_2_ is also a useful method to advance its photocatalysis efficiency through the reduction in the charge carriers’ recombination [19]. Compared with other rare earth metals, Nd is an efficient element that has gained attention in many studies due to its good properties [20,21]. Nd-doped TiO_2_ has the ability to reduce band gaps and overcome the reunion of charges for enhanced photocatalytic activity [22,23,24]. To the best of our knowledge, no report can be found in the literature on Nd and N co-doped TiO_2_ nanomaterials for photocatalytic applications. Therefore, the development of Nd-N/TiO_2_ photocatalytic systems will be a good strategy to achieve a higher decolorization of organic contaminants under visible-light exposure.

In this study, we prepared a Nd-N/TiO_2_ photocatalyst using a simple hydrothermal method. The obtained TiO_2_-based nanomaterials exhibited wider solar light absorption and an enhanced photocatalytic activity in the decomposition of acid blue-29 dye owing to the cooperative effects of the Nd and N atoms in the Nd-N/TiO_2_ system.

## 2. Experimental Procedure

### 2.1. Synthesis of Nd-N/TiO_2_ Photocatalyst by Hydrothermal Method

The Nd-N/TiO_2_ photocatalyst was prepared using a single-step hydrothermal process. In the experiment, the reaction was started by adding the tetrabutyl titanate (TBT, 10 mL) to a water, ethanol and acetic acid mixture (25 mL + 25 mL + 10 mL), which was stirred for 20 min at 25 °C. Following previous steps, the required amount of urea and Nd (NO_3_)_3_ 6H_2_O were mixed into the initial solution. In the next stage, the solution was transported to a stainless-steel autoclave (100 mL) containing a Teflon beaker and was heat treated at 120 °C for 6 h. After the completion of this process, washing of the obtained material was performed using water. In the final step, it was heated at 100 °C for 9 h in an oven initially and then it was treated at 400 °C for 2 h in a furnace. The molar percentage of Ti to urea was 1:2, and in the Nd/TiO_2_ and co-doped sample (Nd-N/TiO_2_), the percentage of the Nd dopant was 2% according to our previous study [23]. The names of the samples were pristine TiO_2_ (obtained without using Nd and N sources), Nd/TiO_2_, N/TiO_2_ and Nd-N/TiO_2_.

### 2.2. Characterization of Synthesized Photocatalysts

In this study, the crystallinity and crystalline phase properties of the prepared materials were examined using a Shimadzu XD-5A powder diffractometer. Transmission electron microscopy (TEM, 2100, Jeol, France) determined the morphology of the nanomaterials. XPS (Thermo-VG Scientific, ESCALAB250, monochromatic) was used for the identification of the chemical states and electronic structure of the photocatalysts. UV–visible absorption spectra were observed with the help of a (UV-1800, Shimadzu, Kyoto, Japan) spectrometer. A fluorescence spectrometer (FP-8200, Jasco, Pfungstadt, Germany) was utilized to obtain PL spectra at a 320 nm wavelength (excitation).

### 2.3. Photocatalytic Efficiency Measurement

To explore the photocatalysis properties of the prepared materials, the degradation of acid blue-29 (AB-29) dye was observed. Its optical absorption maxima occurred at about 630 nm, where the scattering of light was minimum. The experiment was performed in a pyrex glass photoreactor containing water in a circulating case using a halogen lamp (300 W) with a cut-off filter (λ ≥ 420 nm) as the light source. During all the experiments, 100 mg of the photocatalyst material and 10 mg of AB-29 dye was well dispersed in 200 mL of deionized water. Initially, a blank test was accomplished separately for dye degradation without a photocatalyst under light exposure and no appreciable loss of dye was found in the experiment. Moreover, after adding the photocatalyst to the dye solution, the suspension was magnetically stirred for 1 h in the dark to reach adsorption/desorption equilibrium. After the adsorption/desorption equilibrium reaction, the concentration was taken as the initial concentration of the AB-29 dye. Next, under visible light exposure, a photocatalytic experiment was started, and 5 mL of the degraded solution was collected every 30 min to check the degradation of the AB-29 dye using a UV–visible spectrometer (Shimadzu-UV-1800). In addition, every collected 5 mL of solution were centrifuged/filtered and then, after taking UV–visible absorption spectra, they were returned back to the original dye solution immediately.

### 2.4. Antioxidant Activity Measurement (DPPH Assay)

The antioxidant potential of the prepared samples was monitored via the DPPH (2, 2-diphenyl-1-picrylhidrazyl) method following the protocols of Helmy et al. [21]. Initially, the DPPH (1.97 mg) was dissolved in methanol (50 mL) to prepare a stock solution and it was stored at 4 °C to use it further in the experiment. Next, different sample solutions were made by dissolving TiO_2_, Nd/TiO_2_, N/TiO_2_, Nd-N/TiO_2_ and/or ascorbic acid in 1 mL of deionized water to obtain a variety of concentrations (20, 40, 60, 80 and 100 µg/mL) of the catalyst and ascorbic acid. After this, 2 mL of the DPPH solution was mixed with 1 mL of the sample solution containing different concentrations as mentioned above. The mixture of deionized water (1 mL) and DPPH (1 mL) played the role of the control. The concentration of the scavenged radicals was then evaluated via measurements of absorption decrease after 25 min at a 517 nm wavelength. Furthermore, in order to measure the scavenging activity of the DPPH radicals, the following equation was used:

$$Radical\;scavenging\;activity=\frac{\left(Control\;absrobance-Sample\;absorbance\right)\times 100}{Control\;absorbance}$$

where control absorbance means absorbance when the antioxidants are not present, while sample absorbance denotes absorbance when the antioxidants are present in the reaction. All the experiments were repeated three times and the mean value was considered for the experimental results plots.

## 3. Results and Discussion

### 3.1. XRD and TEM Analysis

**Figure 1:**
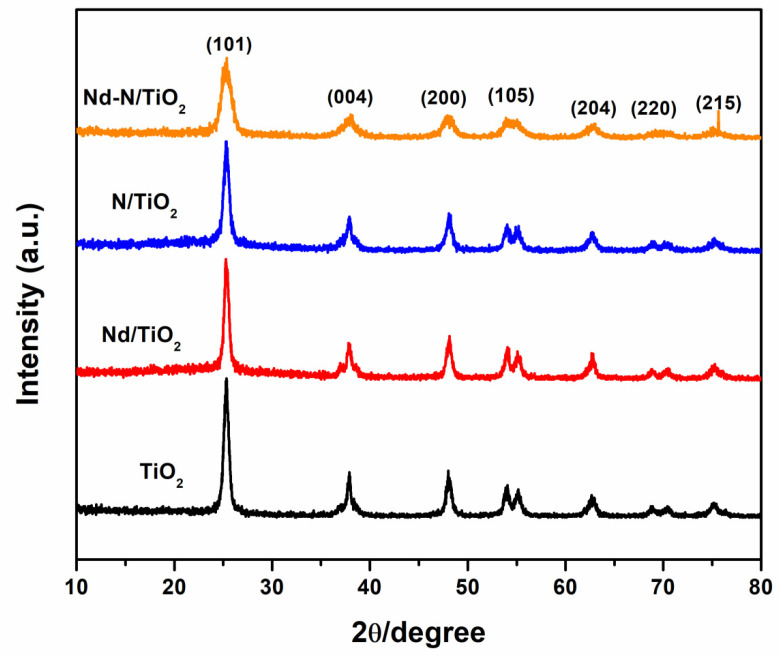
XRD graphs of different photocatalysts prepared by hydrothermal method.

Figure 1 shows the TiO_2_, Nd/TiO_2_, N/TiO_2_ and Nd-N/TiO_2_ nanoparticles’ XRD patterns. The bare TiO_2_ nanoparticles exhibited diffraction peaks at 25.3°, 38.0°, 48°, 54.5°, 62.7°, 69.5° and 75.1°, conforming the values of hkl (101), (004), (200), (105), (204), (220) and (215), respectively. This clearly reveals the anatase crystalline phase of TiO_2_, agreeing with (JCPDS 21-1272). The Nd/TiO_2_, N/TiO_2_ and Nd-N/TiO_2_ samples also showed the same structure (anatase) except the crystallinity seemed to be reduced, indicating that the dopants retarded the transition of the anatase phase to any other phase (rutile or brookite) [25]. Moreover, the absence of Nd or its oxide phase peaks in the Nd/TiO_2_ and Nd-N/TiO_2_ samples might be ascribed to the lower doping contents of Nd or the increased amorphous nature of the doped samples. Moreover, the crystallite size of all the samples was measured using the Scherrer formula [23]. The average crystallite size for the TiO_2_, Nd/TiO_2_, N/TiO_2_ and Nd-N/TiO_2_ samples were found to be 15.4 nm, 13.3 nm, 12.0 nm and 9.6 nm, respectively. This decrease in crystallite size can be attributed to the cooperative effects of Nd and N doping in the TiO_2_ nanoparticles.

**Figure 2:**
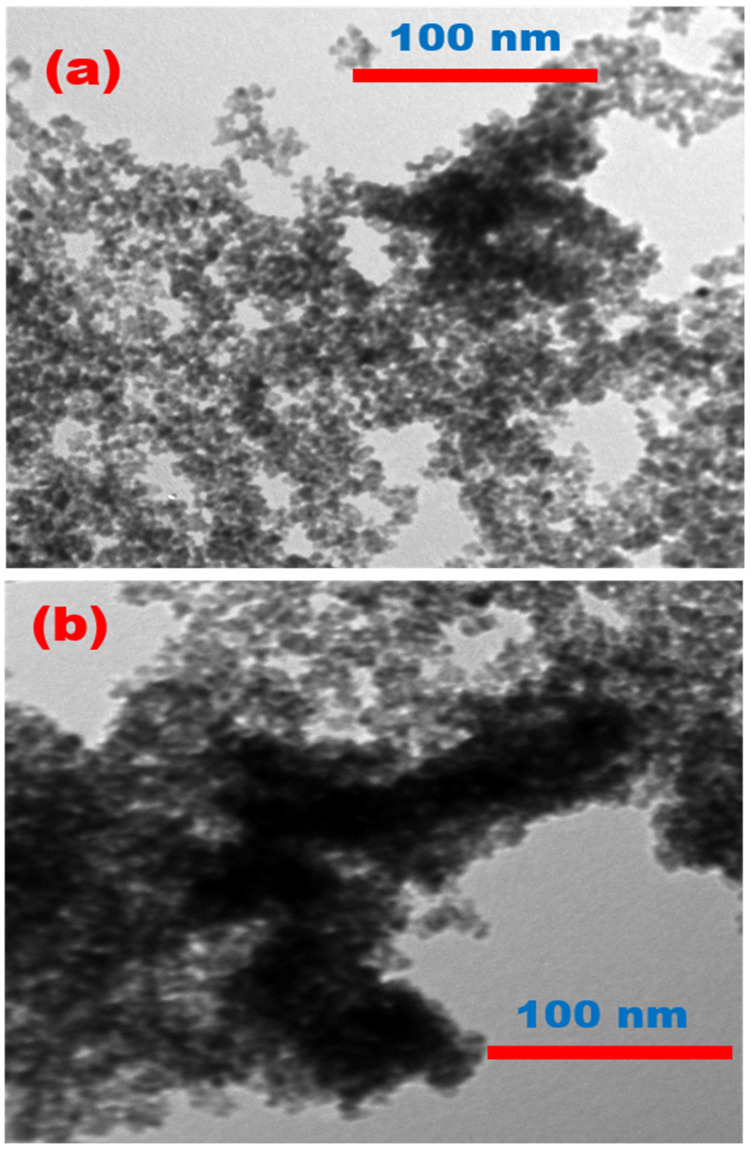
TEM images of (**a**) TiO_2_ and (**b**) Nd-N/TiO_2_ nanoparticles.

TEM studies are useful for identifying the size and shape of nanomaterials. Figure 2a,b illustrate TEM images of the TiO_2_ and Nd-N/TiO_2_ nanomaterials, respectively. The particles in both materials were nearly spherical and were uniformly separated without any distinguishable agglomeration. The average particle size for both materials was less than 20 nm. Furthermore, in the Nd-N/TiO_2_ sample, the black Nd was homogeneously dispersed on the TiO_2_ surface, showing the more defective nature of the Nd-N/TiO_2_ sample.

### 3.2. XPS Analysis

**Figure 3:**
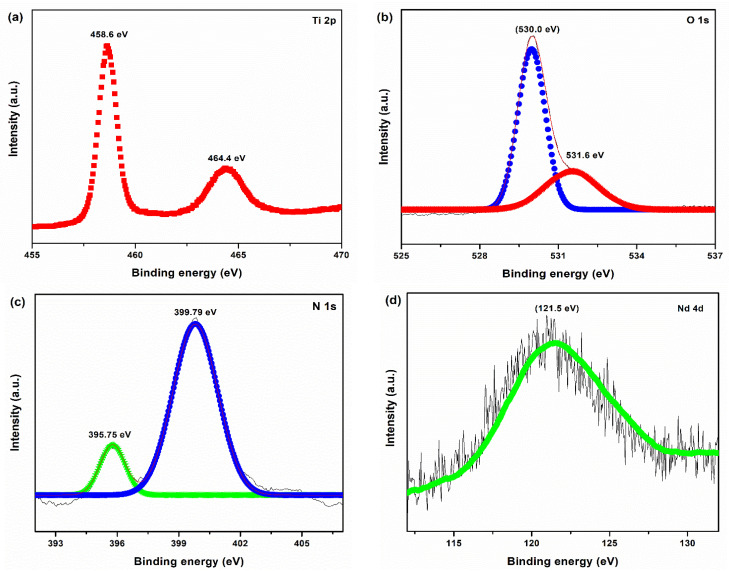
XPS core level spectra of (**a**) Ti 2p, (**b**) O1s, (**c**) N 1s, (**d**) Nd 4d for Nd-N/TiO_2_ sample.

The electronic structure and chemical states of Nd-N/TiO_2_ were studied using XPS. Figure 3 shows the Nd-N/TiO_2_ sample’s XPS spectra of Ti 2p, O 1s, N 1s and Nd 4d. The stronger peaks, namely, Ti 2p_3/2_ and Ti 2p_1/2_, appeared at 458.6 eV and 464.4 eV, respectively (Figure 3a), signifying that titanium metal (Ti) existed as Ti^4+^ [26,27]. As revealed in Figure 3b, the spectrum of O 1s can be fitted into peaks. The appearance of peak at 530.0 eV shows an oxygen atom’s existence in the TiO_2_ lattice (Ti-O bond) and the second peak at 531.6 verifies the presence of a surface hydroxyl oxygen [28]. In a highly resolved spectrum of N 1s, as shown in Figure 3d, the first peak at 395.75 eV can be attributed to Ti-N bonds, which confirm that the doping of N into the TiO_2_ material was substitutional [29]. The other peak at 399.79 verifies the interstitial N doping in the TiO_2_ nanoparticles (Ti-O-N) [30]. In the Nd 4d spectrum (Figure 3d), the single peak appearing at 121.5 eV shows a positive shift of 3.5 eV from the metallic Nd° (118). This positive shift suggests that the Nd dopant was present in the TiO_2_ nanoparticles as Nd^3+^ ions [31,32,33].

### 3.3. UV–Vis Spectroscopy Analysis

**Figure 4:**
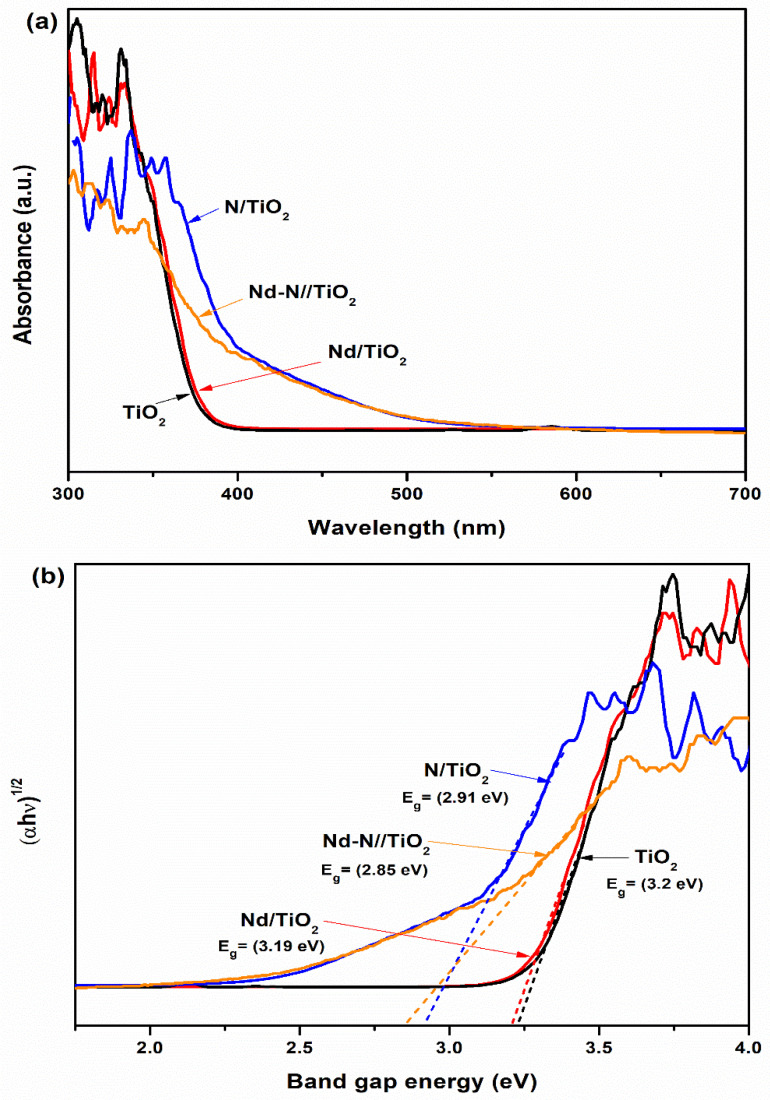
(**a**) Absorption spectra and (**b**) Tauc plots of TiO_2_, Nd/TiO_2_, N/TiO_2_ and Nd-N/TiO_2_ nanoparticles.

Figure 4a shows the absorption spectra of the pristine TiO_2_, Nd/TiO_2_, N/TiO_2_ and Nd-N/TiO_2_ samples. The band gap energy (*E_g_*) of all the samples can be calculated using the equation:

$${\left(\alpha h\vartheta \right)}^{\frac{1}{2}\;}=B\;\left(h\vartheta -E_{g}\right)$$

Here, the absorption coefficient is denoted by ‘α’, while ‘hϑ’ represents the energy of the photon and *B* is a constant [29]. The values of the band gap of the bare TiO_2_, Nd/TiO_2_, N/TiO_2_ and Nd-N/TiO_2_ were calculated to be 3.20 eV, 3.19 eV, 2.91 eV and 2.85 eV, respectively, as shown in Figure 4b. This indicates that the N dopant element had more ability to lessen the band gap of TiO_2_ compared to the Nd dopant. Overall, the doping of Nd and N played an effective role in decreasing the TiO_2_ band gap (3.2 to 2.85 eV). This good reduction in the band gap of the resulting nanomaterial (Nd-N/TiO_2_) was due to the fact that the interactive property of the doping element and TiO_2_ and the creation of new energy states within the TiO_2_ band gap always results in a band gap decrease [32,33].

### 3.4. PL Spectroscopy Analysis

**Figure 5:**
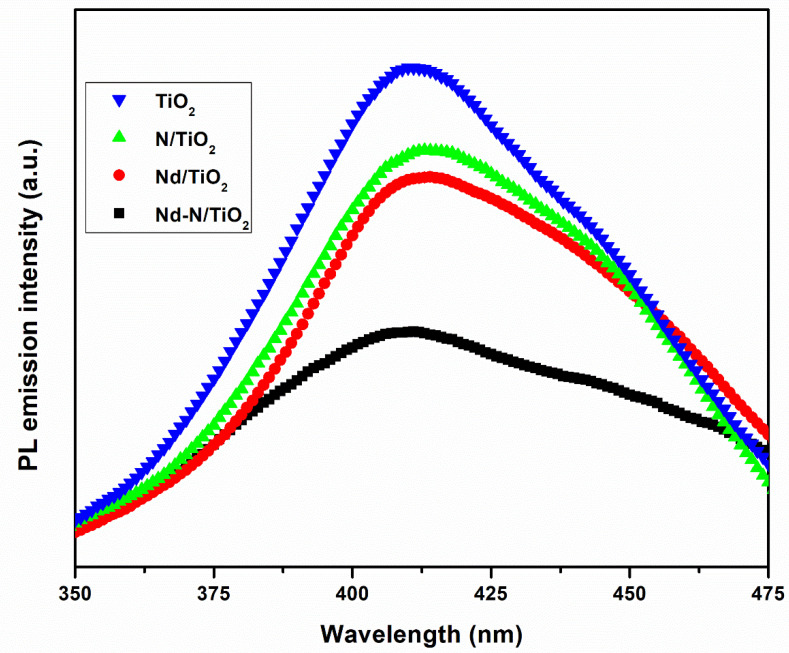
Photoluminescence spectra of TiO_2_, Nd/TiO_2_, N/TiO_2_ and Nd-N/TiO_2_ samples.

PL spectroscopy has been extensively utilized to determine the charge carriers’ trapping efficiency, immigration and transportation to know the destiny of the charges in semiconducting nanomaterials [26]. Figure 5 represents the PL spectra of the TiO_2_, N/TiO_2_, Nd-N/TiO_2_ and Nd-N/TiO_2_ nanoparticles. As displayed in the results, the pristine TiO_2_ had a large PL intensity, whereas, after the incorporation of Nd and N, the PL intensity decreased, displaying a suppressed reunion of the electron/hole. Most interestingly, the co-doped Nd-N/TiO_2_ sample exhibited the lowest PL intensity compared to all the other samples. This is clear evidence that the Nd and N co-doping had a significant influence on separating the charge carriers in TiO_2_ in order to boost photocatalytic performance for dye degradation.

### 3.5. Photocatalytic Activty

**Figure 6:**
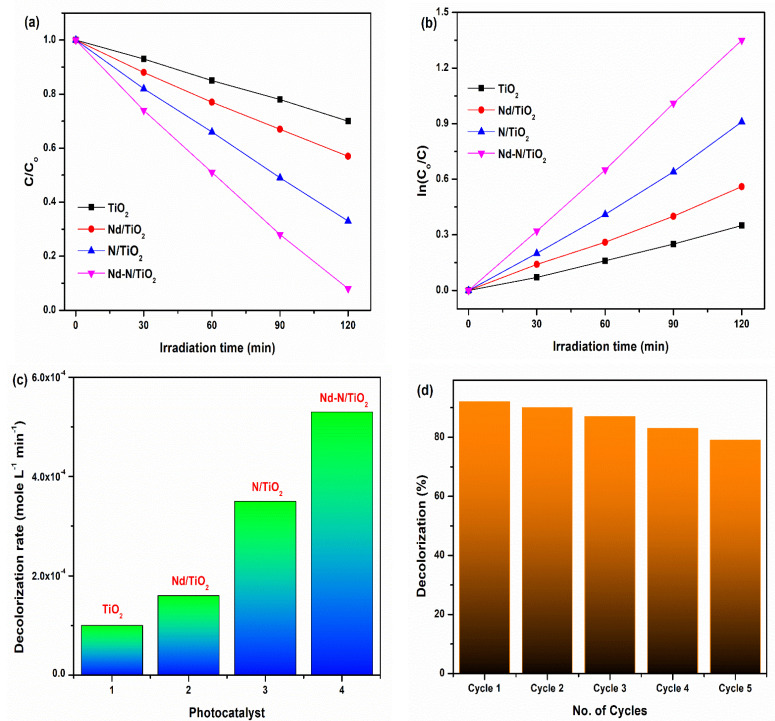
(**a**) Concentration variation (*C*/*C_o_*) of AB-29 dye degradation, (**b**) plots of ln (*C_o_/C*) against exposure time for decolorization of AB-29, (**c**) decolorization rate of AB-29 over TiO_2_, Nd/TiO_2_, N/TiO_2_ and Nd-N/TiO_2_ photocatalysts, (**d**) recyclability of different photocatalysts for five continuous cycles.

The effectiveness of the samples in the decolorization of the AB-29 dye was investigated under the exposure of visible light. Figure 6a shows the relative variation in AB-29 concentration (*C*/*C_o_*) against time using TiO_2_-based photocatalytic nanomaterials. The Nd-N/TiO_2_ photocatalyst demonstrated a 92% degradation of AB-29 dye after 120 min of exposure to solar light, while N/TiO_2_, Nd/TiO_2_ and TiO_2_ only exhibited a 67%, 43% and 31% degradation, respectively. Therefore, it was confirmed that Nd-N/TiO_2_ had better photocatalysis activity than the single-doped (Nd/TiO_2_, N/TiO_2_) or pure TiO_2_. Moreover, Nida et al. [34] found 82% AB-29 dye degradation in an irradiation time of 90 min when they used CdS/TiO_2_ nanoparticles. Most of the dyes during photocatalytic degradation followed the Langmuir–Hinshelwood kinetic model [34,35], and the relation is given as:

$$-\frac{dC}{dt}=\frac{kKC}{\left(1+KC\right)}$$

Here, ‘*k*’ represents the rate constant of the relevant reaction (mM min^−1^), the adsorption coefficient of the reactants is ‘*K*’ (mM^−1^) and *C* denotes the concentration of the reactants (mM). In the case of a low concentration ‘*C*’, one can write the above equation in the form of apparent pseudo-first-order kinetics [35].

$$-\frac{dC}{dt}=kKC=k_{app}C$$

The degradation curve (Figure 6a) represents exponential decay and follows pseudo-first-order kinetics practically well. The rate constant can be found for the samples via a ln (*C_o_*/*C*) plot against illumination time (Figure 6b) using the below equation:

$$\ln(\frac{C_{o}}{C})=k_{app}t$$

where *C_o_* is the staring dye concentration, *C* represents the concentration of the AB-29 dye after time ‘*t*’ and *k_app_* denotes the apparent rate constant (min^−1^). Furthermore, the dye degradation rate was determined using the following formula [36]:

$$\frac{-\left(d\left[C\right]\right)}{dt}\to k{\left[C\right]}^{n}$$

Here, ‘*k*’ denotes the rate constant, *C* represents the dye concentration, while *n* represents the reaction’s order. Figure 6c shows the degradation rate, which followed the order TiO_2_ (1.0 × 10^−4^) < Nd/TiO_2_ (1.6 × 10^−4^) < N/TiO_2_ (3.5 × 10^−4^) < Nd-N/TiO_2_ (5.3 × 10^−4^). The superior photocatalysis efficiency of the Nd-N/TiO_2_ photocatalyst was due to broadening the absorption of solar light and the reduced reunion of the electron/hole, as was confirmed in the UV–vis and PL spectroscopies (Figure 5 and Figure 6).

**Figure 7:**
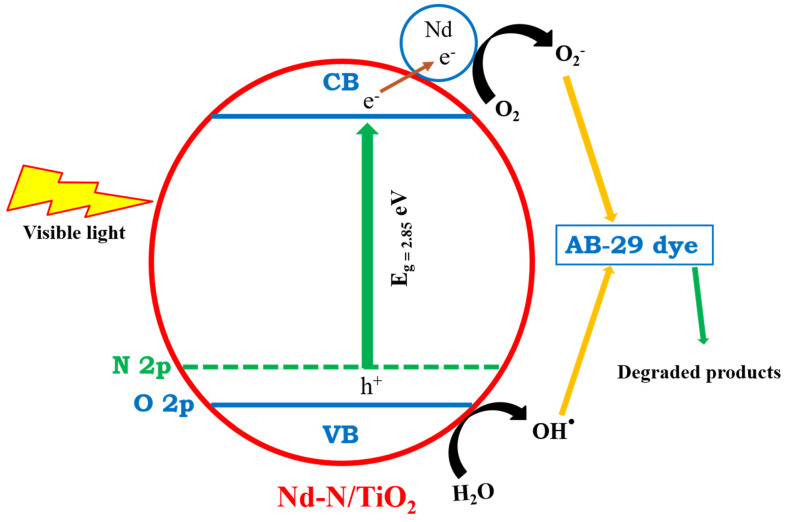
Schematic illustration of photocatalysis mechanism of Nd-N/TiO_2_.

Figure 6d demonstrates the photocatalytic decomposition of the AB-29 dye over the TiO_2_, Nd/TiO_2_, N/TiO_2_ and Nd-N/TiO_2_ nanoparticles over five consecutive cycles. The decrease in the decolorization efficiencies of TiO_2_, Nd/TiO_2_, N/TiO_2_ and Nd-N/TiO_2_ after five cycles was 17%, 18%, 15% and 15%, respectively. This verifies the good stability of the prepared photocatalyst (Nd-N/TO_2_) along with its superior photocatalytic activity. Moreover, the excellent photocatalytic activity of Nd-N/TO_2_ can be understood as indicated in the mechanistic diagram (Figure 7). It is known that rare earth elements were unable to replace Ti^4+^ in the crystal matrix because of their incompatible ionic radii; therefore, rare earth metal ions could exist in the form of rare earth metal oxides and they could be dispersed on the surface of TiO_2_ [37]. Additionally, rare earth metal ions could act as a trapping site, which would efficiently reduce the recombination of the photogenerated charge carriers and then a higher photocatalytic performance could be achieved [28]. Therefore, based on these facts, Nd-N/TiO_2_ (2.85 eV) absorbed visible light photons and, as a result, a higher number of electrons were transported from the valence band to the conduction band of Nd-N/TiO_2_ via Nd^3+^ capturing the sites below the conduction band of the material and participating in the reduction process to produce superoxide radicals (^•^O_2_^−^). At the same time, the holes left behind in the valence band reacted with water to generate hydroxyl radicals (OH^•^). These photogenerated radicals (superoxide and hydroxyl) finally participated in the decomposition of the AB-29 dye when exposed to solar light. Furthermore, as presented in Figure 7, the Nd dopant had the capability to capture electrons from the conduction band of Nd-N/TiO_2_, reducing the reunion of the charges. Thus, in this way, the Nd dopant contributed to enhancing the photocatalytic efficiency of the prepared photocatalyst.

### 3.6. Antioxidant Activity

**Figure 8:**
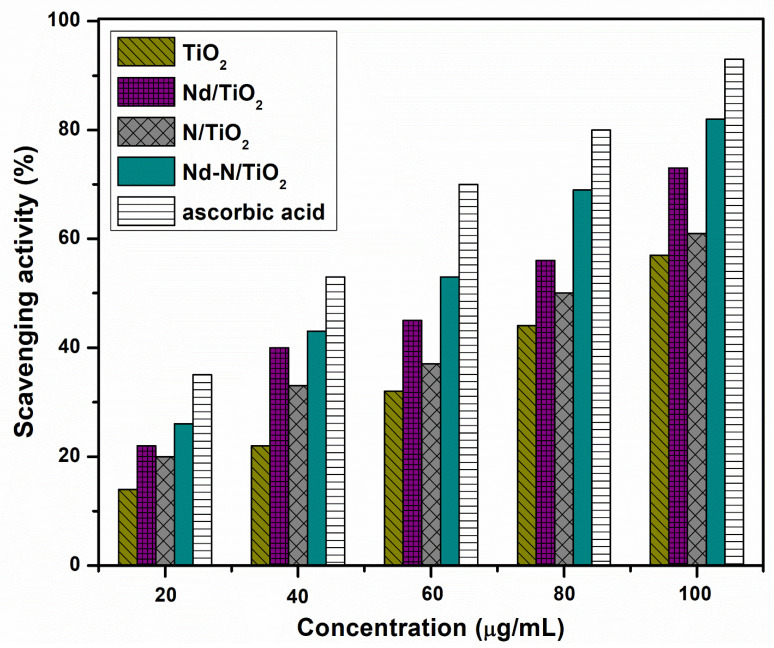
Antioxidant activity of different samples against DPPH assay at different concentrations of nanoparticles in DPPH solution.

The scavenging action of all the samples was assessed via a DPPH test. It is well known that DPPH can be easily converted to a DPPH-H compound (stable) when it accepts H_2_ radicals from the tested nanomaterials [24]. During this process, its color changes from violet (dark) to a dim yellow. Therefore, DPPH reduction gives an estimation of the radical trapping of the tested material. Figure 8 shows the scavenging activity (%) of TiO_2_, N/TiO_2_, Nd-N/TiO_2_ and Nd-N/TiO_2_ for the DPPH assay. The scavenging performance increases with an increasing concentration of the antioxidant. The activity was boosted in the following order: ascorbic acid (35–93%) > Nd-N/TiO_2_ (26–82%) > Nd/TiO_2_ (22–73%) > N/TiO_2_ (20–61%) > TiO_2_ (14–57%), with an increasing concentration from 20 to 100 µg/mL. These results show that Nd-N/TiO_2_ is a superior antioxidant over all other samples. In addition, the results found in this study are good compared with other workers, including plum peals mediated-TiO_2_ nanoparticles, which exhibited a 78% scavenging activity for DPPH at the same concentration (100 µg/mL) [38]. In another report, Hosney et al. [2] observed a 41.89% DPPH scavenging activity at 50 µg/mL when using a Ag/ZnO@Biochar nanocomposite. Rajeswari et al. [39] found a 55% antioxidant activity at 200 µg/mL for DPPH scavenging using a Pd-RGO-ZnO nanocomposite.

## 4. Conclusions

Nd-N/TiO_2_ nanoparticles were synthesized via a simple hydrothermal route. The structural, morphological and optical properties were improved due to Nd and N doping. The incorporation of Nd and N played a greater role in decreasing the band gap of TiO_2_ from 3.20 eV to 2.85 eV, making them able to absorb visible light effectively. In addition, the recombination of the photogenerated charge carriers was greatly reduced due to the co-doping of Nd and N into the Nd-N/TiO_2_ system. Compared with all the other samples, Nd-N/TiO_2_ exhibited a higher photocatalytic efficiency (92%) in the removal of AB-29 dye when irradiated with visible light. Moreover, this novel photocatalyst also indicated a good antioxidant efficiency of 82% at the maximum concentration of nanoparticles in the DPPH solution (100 µg/mL).
